# Adsorption of Cationic Contaminants by Cyclodextrin Nanosponges Cross-Linked with 1,2,3,4-Butanetetracarboxylic Acid and Poly(vinyl alcohol)

**DOI:** 10.3390/polym14020342

**Published:** 2022-01-16

**Authors:** Ekkachai Martwong, Santi Chuetor, Jatupol Junthip

**Affiliations:** 1 Division of Science (Chemistry), Faculty of Science and Technology, Rajamangala University of Technology Suvarnabhumi, Phra Nakhon Si Ayutthaya 13000, Thailand; ekkachai.m@rmutsb.ac.th; 2 Department of Chemical Engineering, Faculty of Engineering, King Mongkut’s University of Technology North Bangkok, Bangkok 10800, Thailand; santi.c@eng.kmutnb.ac.th; 3 Faculty of Science and Technology, Nakhon Ratchasima Rajabhat University, Nakhon Ratchasima 30000, Thailand

**Keywords:** paraquat, malachite green, safranin, adsorption, nanosponges, cyclodextrin, 1,2,3,4-butanetetracarboxylic acid, poly(vinyl alcohol), wastewater treatment, pseudo-second-order, Langmuir isotherm

## Abstract

Cationic organic pollutants (dyes and pesticides) are mainly hydrosoluble and easily contaminate water and create a serious problem for biotic and abiotic species. The elimination of these dangerous contaminants from water was accomplished by adsorption using cyclodextrin nanosponges. These nanosponges were elaborated by the cross-linking between 1,2,3,4-butanetetracarboxylic acid and β-cyclodextrin in the presence of poly(vinyl alcohol). Their physicochemical characteristics were characterized by gravimetry, acid-base titration, TGA, ^13^C NMR, ATR-FTIR, Raman, X-ray diffraction, and Stereomicroscopy. The BP5 nanosponges displayed 68.4% yield, 3.31 mmol/g COOH groups, 0.16 mmol/g β-CD content, 54.2% swelling, 97.0% PQ removal, 96.7% SO removal, and 98.3% MG removal for 25 mg/L of initial concentration. The pseudo-second-order model was suitable for kinetics using 180 min of contact time. Langmuir isotherm was suitable for isotherm with the maximum adsorption of 120.5, 92.6, and 64.9 mg/g for paraquat (PQ), safranin (SO), and malachite green (MG) adsorption, respectively. Finally, the reusability performance after five regeneration times reached 94.1%, 91.6%, and 94.6% for PQ, SO, and MG adsorption, respectively.

## 1. Introduction

Water pollution contaminated by dyes and pesticides is still a recent issue that has intimidated both the ecosystem and human health. The adsorption technique provides essential advantages for wastewater treatment such as high efficiency, design simplicity, ease of operation, low-cost process, reusability of adsorbents, selectivity of adsorbents, and flexibility for industry technology transfer. This adsorption process could remove any cationic, anionic, or neutral pollutants by innovative adsorbents.

Paraquat (PQ) is a water-soluble agrochemical that is classified as a non-selective herbicide for plantation or defoliation. This herbicide threatens both the environment [1,2] and health [3,4,5]. Biological [6,7], physical [8,9], and chemical treatments [10,11] have all been informed in the previous studies for PQ removal. Different effective adsorbents were elaborated for PQ removal such as activated carbon [12], bentonite [13], bio-based material [14,15,16], carbon nanotubes [17], calixarene [18], cellulose nanofiber [19], cyclodextrin [20,21], graphene oxide [22], kaolin [23], magnetic adsorbent [24,25], microorganisms [26], montmorillonite [27], pillararene [28], and silica [29,30].

Malachite green (MG) is a hydrosoluble cationic dye [31] with a chemical structure of triphenylmethane that is used as an antiparasitic, a fungicide, a parasiticide, an anthelminthic a disinfectant, a coloring agent and additive, and a candidate for the endocrine disruptive compound. This colorant is widely used in various applications such as textile, paper, leather, pharmaceutical, and aquacultural industries. Because of its high solubility and stability in water, the trace of these synthetic organic dyes produced important public health hazards (carcinogenic and mutagenic effects) [32] and caused potential environmental problems [33,34]. Biological [35,36,37], physical [38,39] and chemical [40,41] treatments have all been informed in the previous studies for MG removal. Therefore, MG was reported in the literature by adsorption [42,43]. Various adsorbents were prepared for MG removal such as activated carbon [31,44], bentonite [45,46], bio-based material [47,48,49], carbon nanotubes [50], cellulose nanofiber [51,52], chitosan [53], clay [54], cyclodextrin [55,56], graphene oxide [57], kaolin [58,59], magnetic adsorbent [60,61], metal-organic framework [62], microorganisms [35,36,37], montmorillonite [63,64], and silica [65,66,67].

Safranin (SO) is a water-soluble cationic dye that displays as a reddish-brown powder. This azine dye was used as a colorant in the textile industry for fabric coloration, the food industry for food coloration, and the medical field for staining bacteria. SO has affected public health and the environment [68]. Biological [69,70], physical [71,72], and chemical [73,74] treatments have all been informed in the previous studies for PQ removal. Therefore, SO was reported in the literature by adsorption. Various adsorbents were prepared for SO removal such as activated carbon [75], bentonite [76], bio-based material [77,78], carbon nanotubes [79], cellulose nanofiber [80], cyclodextrin [81,82], copper oxide nanoparticles [83], graphene oxide [84], kaolin [85,86], magnetic adsorbent [87], metal-organic framework [88], microorganisms [69,70], montmorillonite [89], and silica [90,91].

β-cyclodextrin (β-CD) contains seven units of d-glucose joined by α-(1,4) glycosidic linkages, which shows a well-defined cyclic structure with a hydrophobic cavity and a hydrophilic exterior. The extraordinary characteristics of this supramolecular molecule provide the encapsulation of organic components with a suitable size into the cyclodextrin cavity by host-guest interaction to form an inclusion complex. Cyclodextrin polymers have widely been developed by different types of polymerization to enhance the solubility for both the guest molecule and the cyclodextrin polymer and improve the adsorption capacity towards organic molecules. Adsorbents based on cyclodextrin and its derivatives have been widely used for environmental applications [20,92,93,94,95,96,97,98,99,100,101,102,103,104,105,106,107,108,109,110,111,112,113,114] and other fields [115,116,117,118,119,120]. Moreover, insoluble cyclodextrin polymers are also named “cyclodextrin nanosponges” because these nanomaterials exhibit a hyperbranched framework with a three-dimensional structure, a sponge-like pattern, and a specific mission to react with a functional cross-linker or other chemical reactants [95].

1,2,3,4-butanetetracarboxylic acid and (BTCA) was used as a tetrafunctional cross-linking agent to elaborate the cross-linked network, which showed meanwhile an anionic feature by the presence of non-reticulated carboxylic groups. Cyclodextrin nanosponges bridged with BTCA were studied in the literature for the adsorption of bisphenol A [121], radionuclides (Uranium (VI) and Europium (III)) [122], and paraquat [21] from aqueous solution. Poly(vinyl alcohol) is a semi-crystalline, synthetic, and water-soluble polymer. This linear polymer displays an exceptional degree of swelling, biodegradability, nontoxicity, and good mechanical properties that can also esterify with BTCA or other cross-linkers to fabricate remarkable insoluble three-dimensional polymers. Thus, cyclodextrin nanosponges containing poly(vinyl alcohol) could boost the adsorption capacity towards organic contaminant molecules because the unoccupied hydroxyl functions on the polymeric chains could represent adsorption sites to attract these molecules through hydrogen bonding [123].

However, cyclodextrin nanosponges cross-linked with BTCA in presence of PVOH have never been declared for the adsorption with these three soluble cationic contaminants (PQ, SO, and MG), which was the criteria for compound selection. In this study, these nanosponges were first elaborated by the cross-linking of BTCA with β-CD and/or PVOH, and their physicochemical characteristics were investigated by various techniques. The kinetics, isotherm, and the recyclability of pollutant adsorption were then studied.

## 2. Materials and Methods

### 2.1. Materials

β-cyclodextrin (Acros Organics, Geel, Belgium), 1,2,3,4-butanetetracarboxylic acid (Acros Organics, Geel, Belgium), Poly(vinyl alcohol) M_w_ = 89,000–98,000 with 99+% hydrolyzed (Sigma-Aldrich, Saint Louis, MO, USA), sodium hypophosphite (Acros Organics, Geel, Belgium), paraquat dichloride hydrate (Sigma-Aldrich, Saint Louis, MO, USA), safranin (S.D. Fine Chem Ltd., Mumbai, India), and malachite green (Riedel-de Haën, Seelze, Germany) were obtained from commercial sources. Other chemicals were analytical grade and ultrapure water was employed for all experiments.

### 2.2. Nanosponges Preparation

The nanosponges were synthesized by the previous method [21]. The mixture containing 10% *w/v* β-CD, 11.14% *w/v* BTCA, 3% *w/v* sodium hypophosphite and the different compositions of PVOH (0.5, 2, or 5% *w*/*v*) was dissolved in 100 mL of water and heated under magnetic agitation. This was then placed into a rotary evaporator at 70 °C to completely eliminate water under vacuum until a solid mixture was achieved, after which it was cross-linked in a rotary evaporator (Heidolph Hei-VAP Advantage, Schwabach, Germany) at 180 °C for 30 min under vacuum. The nanosponges were rinsed with water and ethanol before submitting at 120 °C to eliminate all of the solvents. Finally, the fine powder of nanosponges was received after crushing with a mortar and a pestle.

### 2.3. Nanosponges Characterization

The physicochemical properties of nanosponges were characterized by different techniques. The reaction yield was found by the proportion between the product mass and the reactant mass. The thermogravimetric analysis (TGA) experiments were operated in an alumina pan with a Thermal Analyzer—STA 449 F3 (NETZSCH, Waldkraiburg, Germany) with a heating rate of 10 °C min^−^^1^ under nitrogen. The morphology of nanosponges was recorded by a SMZ745T stereomicroscope (Nikon, Melville, NY, USA) equipped with a DS-Fi3 digital camera. Raman spectroscopy experiments were run on a Cora 5700 Raman spectrometer (Anton Parr, Bangkok, Thailand) using 300 mW of laser power, 785 nm of laser wavelength, 10 s of integration time, and 100–2000 cm^−1^ of spectral range, with a resolution of 9 cm^−1^. Fourier transform infrared spectroscopy (FTIR) experiments using attenuated total reflection (ATR) mode were executed on a Tensor 27 FTIR (Bruker, Billerica, MA, USA), which accumulated from 64 scans in the 700–4000 cm^−^^1^ range with a resolution of 4 cm^−^^1^. X-ray Diffraction (XRD) spectra were registered on a SmartLab SE X-ray diffractometer (Rigaku, Tokyo, Japan) using an angular range (2θ) between 10° to 70°, 10°/min of scan speed, 0.02° of step width of 0.02°, 40 kV of generator voltage, and 50 mA of generator current.^13^C NMR (nuclear magnetic resonance) spectra were performed on an Ascend 400 WB spectrometer (Bruker, Billerica, MA, USA) at 100.62 MHz and 298 K using the magic angle spinning (MAS) technique, glycine as a reference, a delay time of 8 s, and a contact time of 1.5 ms.

The determination of the β-CD cavities of the nanosponges was performed by photometric titration using phenolphthalein. A total of 20 mg of nanosponges was added into a 25 mL of volumetric flask containing 1 mL of phenolphthalein (0.68 mmol L^−^^1^), 2.5 mL of Na_2_CO_3_ (1 mol L^−^^1^), and 21.5 mL of water. After shaking (150 rpm) for 24 h, the aliquot was measured at 552 nm by a GENESYS 10S UV-Vis spectrophotometer (Thermo Scientific, Vantaa, Finland). The β-CD content was expressed in mmol per gram of nanosponges using the calibration curve which was created by replacing nanosponges with different mass of β-CD (2, 4, 6, 8, and 10 mg).

The measurement of the ion exchange capacity (IEC) of nanosponges was executed by pH-metric titration. The nanosponges (0.1 g) were dipped into 50 mL of 2% *w/v* calcium acetate solution for 4 h under agitation at 150 rpm. After removing the sample, the solution containing acetic acid was titrated by NaOH solution (0.05 M) using phenolphthalein as an indicator. The IEC was expressed in mmol of COOH groups per gram of nanosponges using the following equation:

(1) $$\mathrm{IEC}\;(\mathrm{mmol}/g)=\frac{C_{\mathrm{NaOH}\;}\times {\;V}_{\mathrm{NaOH}}}{m}\;$$

where V_NaOH_ and C_NaOH_ correspond, respectively, to the equivalent volume (mL) and concentration (mol/L) of NaOH. The symbol m refers to nanosponges weight (g). Experiments were operated in triplicate.

The swelling behavior of the nanosponges was studied by solution uptake determination. Ultrapure water (10 mL) was added to a test tube containing 100 mg of nanosponges was added to a test tube containing 10 mL of water. After 24 h of immersion under agitation of 150 rpm, the swollen nanosponges were entirely drained before weighing. The swelling was calculated in percent using the following equation:

(2) $$\mathrm{Swelling}\;\left(\%\right)=\frac{W_{2}-W_{1}}{W_{1}}\times 100\;$$

where W_1_ and W_2_ refer, respectively, to dried and swollen nanosponges. Experiments were executed in triplicate.

### 2.4. Adsorption Study

#### 2.4.1. Preliminary Adsorption Study

An amount of 10 mL of contaminant solution (PQ, SO, or MG) with a 25 mg/L of initial concentration at different pH (2, 3, 4, 5, 6, 7, 8, 9, and 10), which was adjusted with 0.1 M HCl and 0.1 M NaOH, was added to a test tube containing 20 mg of nanosponges under stirring (150 rpm) for 180 min at 30 °C. The amount of contaminant was quantified by UV-Vis spectrophotometer (GENESYS 10S, Thermo Scientific, Vantaa, Finland) at 257 nm, 520 nm, or 619 nm for PQ, SO, or MG respectively. The removal was expressed in percentage using the following equation:

(3) $$\%\;\mathrm{Removal}\;=\frac{{(C}_{0}-C_{t})}{C_{0}}\times 100$$

where C_0_ and C_t_ relate, respectively, to the initial and real-time concentration of the contaminant. Experiments were performed in triplicate. The adsorption capacity (Q) was also exhibited using the following equation:

(4) $$\mathrm{Adsorption}\;\mathrm{capacity}\;\left(\mathrm{mg}/g\right)=\frac{{(C}_{0}-C_{t})\;\times \;V}{m}$$

where C_0_ and C_t_ relate, respectively, to the initial and real-time concentration of the contaminant, V refers to solution volume, and m stands for nanosponges mass.

#### 2.4.2. Kinetics Study

An amount of 10 mL of contaminant solution with a 25 mg/L initial concentration and optimal pH was poured into a test tube containing 20 mg of nanosponges under agitation of 150 rpm at different times (15, 30, 45, 60, 120, 180, and 300 min) at 30 °C. The measurement of contaminants was described in the previous section. Experimental data were then fitted with two kinetics models:

Pseudo-first-order model:

ln (Q_e_ − Q_t_) = ln Q_e_ − k_1_t (5)

Pseudo-second-order model:

(6) $$\frac{t}{Q_{t}}=\frac{1}{k_{2}Q_{e}^{2}}+\frac{1}{Q_{e}}t$$

where Q_e_ and Q_t_ are the quantity of contaminant adsorbed (in mg/g) at equilibrium and at time t, respectively, k_1_ (/min) and k_2_ (g/mg·min) are adsorption rate constant, and t is contact time (min). Experiments were performed in triplicate.

The quantity of contaminant adsorbed versus the square root of time was plotted using the intraparticle diffusion model as the following equation:

Intraparticle diffusion model:

Q_t_ = k_3i_t^0.5^ (7)

where Q_t_ are the quantity of contaminant adsorbed (in mg/g) at time t, respectively, k_3i_ (g/mg·min^0.5^) is adsorption rate constant, and t is contact time (min). Experiments were performed in triplicate.

#### 2.4.3. Isotherm Study

An amount of 10 mL of contaminant solution with different initial concentrations (25, 50, 150, 250, and 300 mg/L) and optimal pH was poured into a test tube containing 20 mg of nanosponges under agitation of 150 rpm at equilibrium and 30 °C. The quantification of PQ was previously mentioned. Experimental data were then fitted with two isotherm models:

Langmuir isotherm:

(8) $$\frac{C_{e}}{Q_{e}}=\frac{1}{K_{L}Q_{m}}+\frac{C_{e}}{Q_{m}}$$

Freundlich isotherm:

(9) $$\ln\;\mathrm{Qe}\;={\;\ln\;K}_{F}+\frac{1}{n}\ln\;\mathrm{Ce}$$

where C_e_ is the equilibrium concentration of contaminant, Q_e_ is the amount of contaminant adsorbed (in mg/g) at equilibrium, Q_m_ is the theoretical maximum adsorption capacity (in mg/g), K_L_ is the Langmuir isotherm constant, K_F_ is the Freundlich isotherm constant, and 1/n is heterogeneity factor.

The Chi-square test was also used as a statistical analysis so as to access the suitability of isotherm equations to the experimental data. The Chi-square value (χ^2^) was expressed by the following equation:

Chi-square value:

(10) $$\chi^{2}=\sum^{\;}\frac{{{(Q}_{e,\exp}{-\;Q}_{e,\mathrm{cal}})}^{2}}{Q_{e,\mathrm{cal}}\;}$$

where Q_e,exp_ is the amount of contaminant adsorbed (in mg/g) at equilibrium calculated from the experimental data and Q_e,cal_ is the amount of contaminant adsorbed (in mg/g) at equilibrium estimated from the models.

#### 2.4.4. Reusability Study

An amount of 10 mL of contaminant solution with 25 mg/L of initial concentration and optimal pH was poured into a test tube containing 20 mg of nanosponges under agitation of 150 rpm at equilibrium and 30 °C. The measurement of contaminants was described in the previous section. However, the adsorbent was then separated and regenerated by cleaning in methanol for PQ desorption and in 5% *v/v* of HCI in ethanol for SO and MG desorption. After 180 min of soaking, the adsorbent was washed with ultrapure water and reconditioned for sorption in posterior cycles.

## 3. Results and Discussion

### 3.1. Synthesis and Characterization of Nanosponges

#### 3.1.1. Physicochemical Properties of Nanosponges

**Figure 1:**
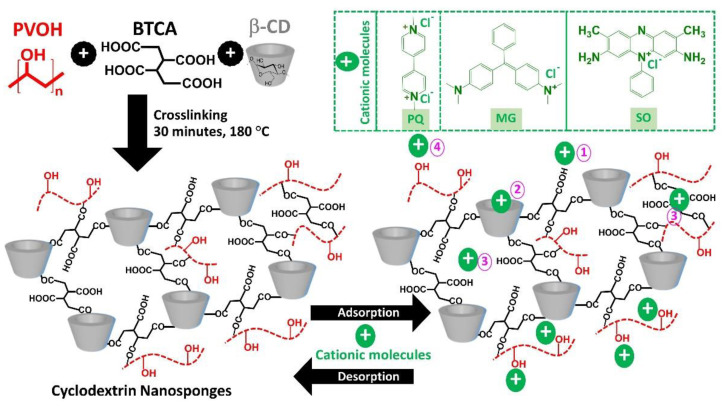
Schematic reaction of cyclodextrin nanosponges and the possible adsorption mechanism with cationic contaminants.

The cyclodextrin nanosponges were successfully elaborated by cross-linking between β-CD and BTCA in the presence of PVOH through esterification reaction to produce negative charges according to the presence of uncross-linked carboxylic groups from BTCA, which could be changed into carboxylate functions, as shown in Figure 1. The cross-linked structure of nanosponges was obtained from the polyaddition between various reactants to establish the various skeletons such as BTCA reticulated with β-CD forms, BTCA reticulated with PVOH forms, and BTCA reticulated with PVOH and β-CD forms. Moreover, the free COOH groups from these three previous skeletons could be newly bridged with β-CD and/or PVOH. The attachment of PVOH at the end of the polymeric segment was extremely useful to enhance the adsorption of various contaminants by hydrogen bonding on the PVOH part. Therefore, these nanosponges displayed an anionic character with the supplementary role of PVOH to eradicate cationic pollutants from wastewater.

As observed in Table 1, for the nanosponges named BP0.5, BP2, and BP5, the addition of PVOH reduced both the reaction yield (from 73.3% to 68.4%) and the ion exchange capacity (from 3.69 mmol/g to 3.31 mmol/g). The esterification between the OH groups of PVOH and the available COOH functions of BTCA could contest with the main reaction between the OH groups of β-CD and the available COOH functions of BTCA to create the cross-linked structure which decreased the reaction yield, as reported in the literature using citric acid as cross-linking agent [124]. Consequently, the number of independent COOH functions was reduced with a higher quantity of PVOH which also decreased the ion exchange capacity. Furthermore, the swelling dropped from 63.5% to 54.2% with an increase in the amount of PVOH because the robust cross-linking density of new ester bridges in the polymer framework was obtained from the polycondensation between PVOH and BTCA, which conducted the nanosponges more packed, prohibited the movement of polymer chains, and prevent the insertion of water into the polymer network.

Astonishingly, the enhance of β-CD content with an increase in PVOH was observed from 0.085 mmol/g to 0.160 mmol/g. The small values of β-CD content were remarked for all systems of nanosponges because the solidity of the cross-linked structure hindered sterically the accessibility of phenolphthalein into cyclodextrin cavities. However, a rise of β-CD content with an augmentation of PVOH may occur according to the adsorption of phenolphthalein on the reticulated PVOH network.

#### 3.1.2. TGA Analysis

**Figure 2:**
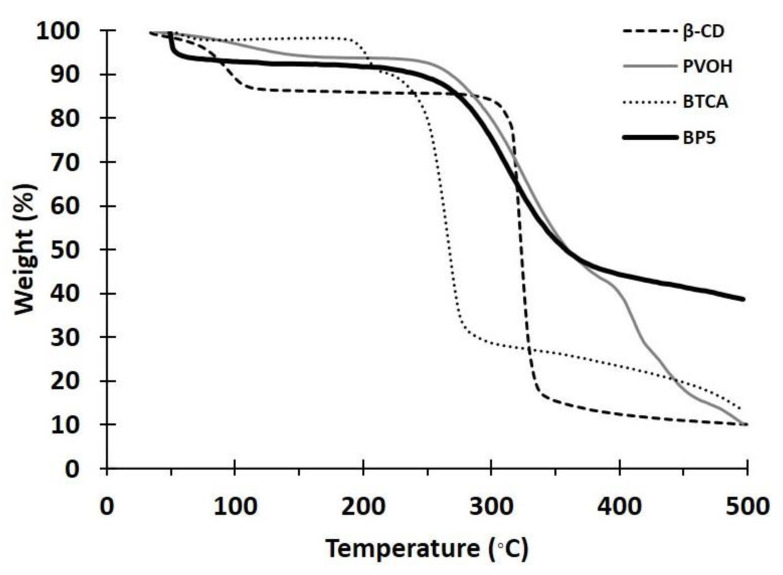
TGA thermograms of BTCA, PVOH, BP5, and β-CD.

The thermal stability of the nanosponges was investigated by TGA, as displayed in Figure 2, for BTCA, PVOH, BP5, and β-CD. The loss of mass below 100 °C referred to the dehydration of samples, which was equal to 2.1%, 2.9%, 8.5%, and 10.7%, respectively. The thermal degradation then began at 193 °C, 241 °C, 206 °C, and 296 °C, respectively. For BP5, the smooth degradation of a residue above 500 °C was thermally stable with the remaining weight of 38.6%.

#### 3.1.3. ATR-FTIR Exploration

**Figure 3:**
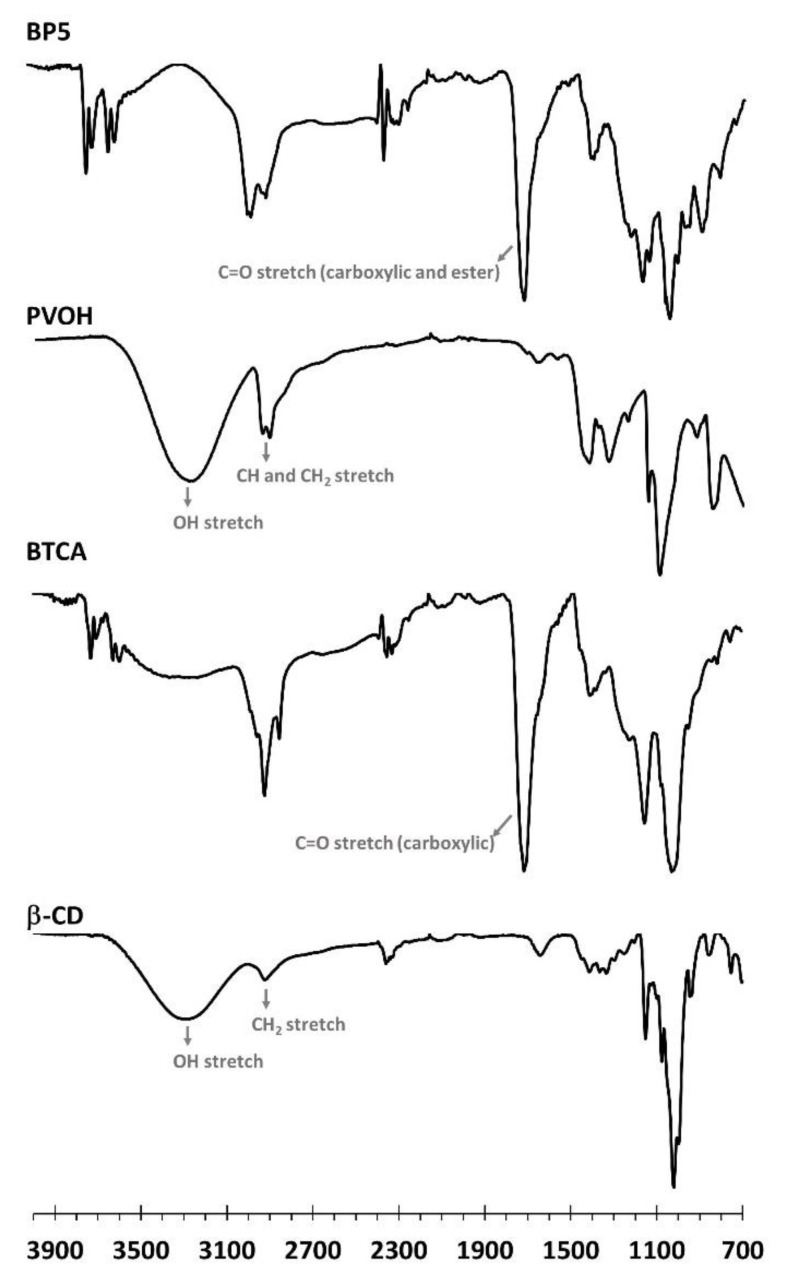
ATR-FTIR spectra of β-CD, BTCA, PVOH, and BP5 nanosponges.

In Figure 3, the functional groups shown on nanosponges were characterized by ATR-FTIR. The native β-CD spectra revealed exclusive specific peaks at 3288 cm^−1^, attributed to OH stretching, at 2917 cm^−1^, attributed to CH_2_ stretching, and at 1152 cm^−1^, attributed to C–O–C stretching of the glycosidic bond, which is in agreement with the data reported in the literature [125]. The BTCA spectra showed particular peaks at 3310 cm^−1^, attributed to OH stretching, at 2923 cm^−1^, attributed to CH stretching, at 2850 cm^−1^, attributed to CH_2_ stretching, and at 1712 cm^−1^, attributed to C=O stretching of carboxylic functions, as informed in previous works [126]. The PVOH spectra displayed specific peaks at 3267 cm^−1^, attributed to OH stretching, at 2939 cm^−1^, attributed to CH stretching, at 2907 cm^−1^, attributed to CH_2_ stretching, at 1417 cm^−1^, attributed to CH bending, and at 1087 cm^−1^, attributed to C–O–C stretching. The characteristic peak at 1715 cm^−1^ for BP5 was attributed to C=O stretching of carboxylic and ester functions, which were superposed to each other. So, the formation of ester bonds confirmed the polyaddition between COOH groups from the BTCA and OH groups forming β-CD and/or PVOH, as notified in antecedent work [127].

#### 3.1.4. Raman Investigation

**Figure 4:**
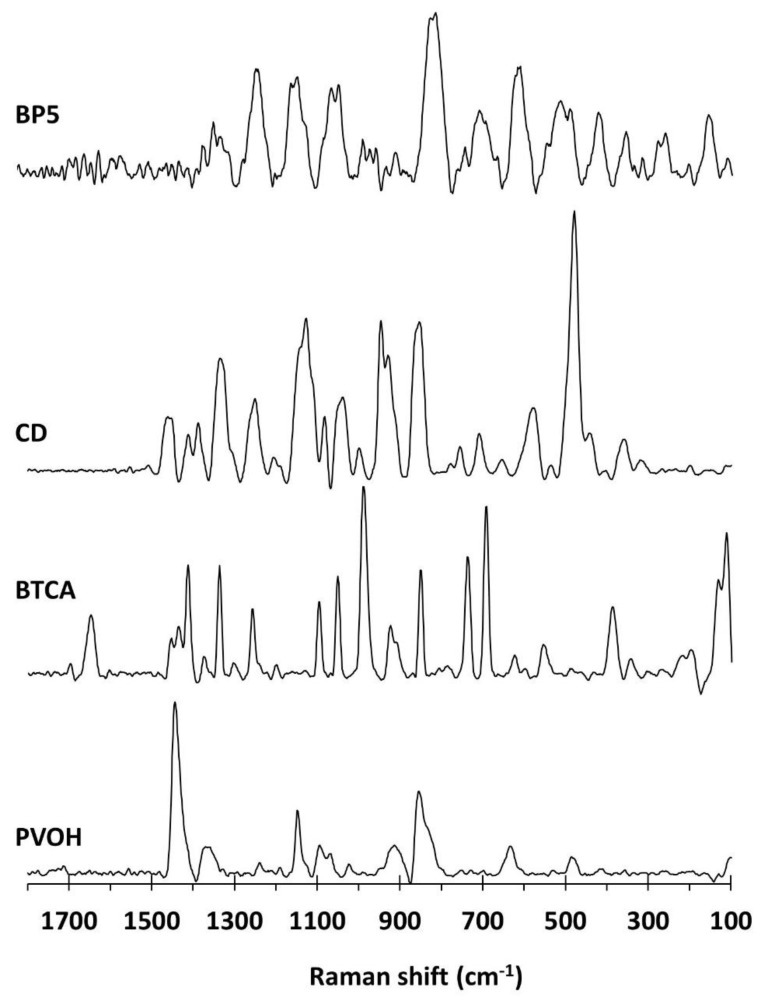
Raman spectra of β-CD, BTCA, PVOH, and BP5 nanosponges.

Raman spectroscopy was carried out to identify the functional groups presented on nanosponges, as illustrated in Figure 4. The PVOH spectra indicated the distinct peaks at 1445 cm^−1^ for CH bending and OH bending, 1376 cm^−1^ for CH bending and OH bending, 1146 cm^−1^ for C–C and C–O stretching, 1097 cm^−1^ for C–O stretching and OH bending, 918 cm^−1^ for C–C stretching, and 857 cm^−1^ for C–C–O stretching, as informed in previous works [128,129,130,131]. The BTCA spectra showed the characteristic particular peaks at 1650 cm^−1^ for C=O stretching of the carboxylic groups, as reported in a previous study [132]. The β-CD spectra exhibited the specific bands at 1463 cm^−1^ for CH deformation, 1338 cm^−1^ for CH_2_ deformation, 1252 cm^−1^ for OH in-plane bending and CH_2_ stretching, 1125 cm^−1^ for C–O–C symmetric stretching, 1081 cm^−1^ for C–O–C symmetric and antisymmetric stretching of glycosidic bonds, and 479 cm^−1^ for skeletal vibrations of amylose. These results were in agreement with the literature [133,134,135,136]. For BP5 nanosponges, the particular peaks were found at 1229 cm^−1^ for CH_2_ stretching, 1144 cm^−1^ for CC and CO stretching, 1043 cm^−1^ for C–O stretching, and 810 cm^−1^ for C–O–C stretching. Nevertheless, the expected peak near 1650 cm^−1^ for C=O stretching of the carboxylic and ester functions was not appeared outstandingly, which might be superposed with the baseline.

#### 3.1.5. NMR Characterization

**Figure 5:**
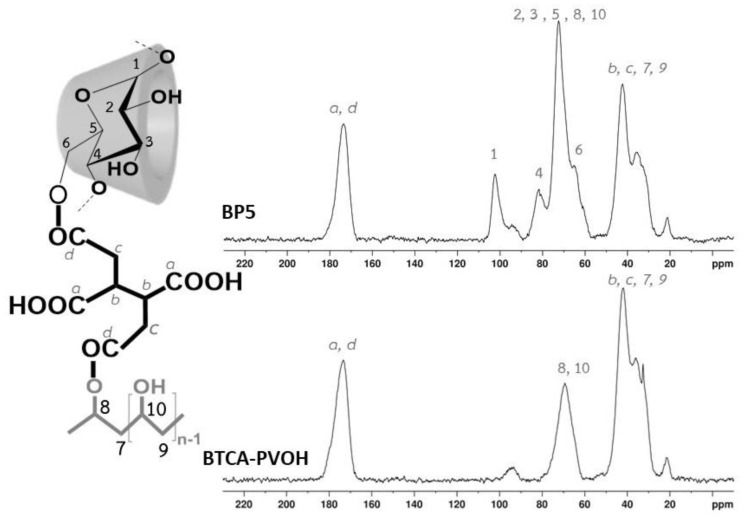
^13^C NMR spectra of BTCA-PVOH and BP5 nanosponges.

Samples were characterized by ^13^C NMR spectroscopy to observe the chemical structure before and after polycondensation. The chemical shift of various reactants (spectra not shown) was similarly described in the nanosponges as seen in Figure 5: PVOH (at 44.5 ppm (for 10) and 70.3 ppm (for 9)), β-CD (at 60.7 ppm (for 6), 74.9 ppm (for 2 and 5), 78.1 ppm (for 3), 82.9 ppm (for 4), and 103.0 ppm (for 1)), and BTCA (at 32.5 ppm (for c), 41.6 ppm (for b), 172.9 ppm (for d), and 173.5 ppm (for a)).

To identify BP5 nanosponges, the sample BTCA-PVOH was also prepared without CD which displayed particular peaks at 173.3 ppm (for a and d), 69.3 ppm (for 8 and 10, and c), and 41.9 ppm (for b,c, 7, and 9). The change of chemical shift also proved the polycondensation between BTCA and PVOH. The ^13^C spectra of BP5 exhibited characteristic peaks at 173.6 ppm (for a and d), 102.1 ppm (for 1), 81.6 ppm (for 4), 72.4 ppm (for 2, 3, 5, 8, and 10), 64.9 ppm (for 6), and 42.3 ppm (for b,c, 7, and 9). Therefore, the esterification of BTCA and β-CD or BTCA and PVOH were confirmed by the slight change of NMR position, as reported [21,137,138] in the literature.

#### 3.1.6. XRD Measurement

**Figure 6:**
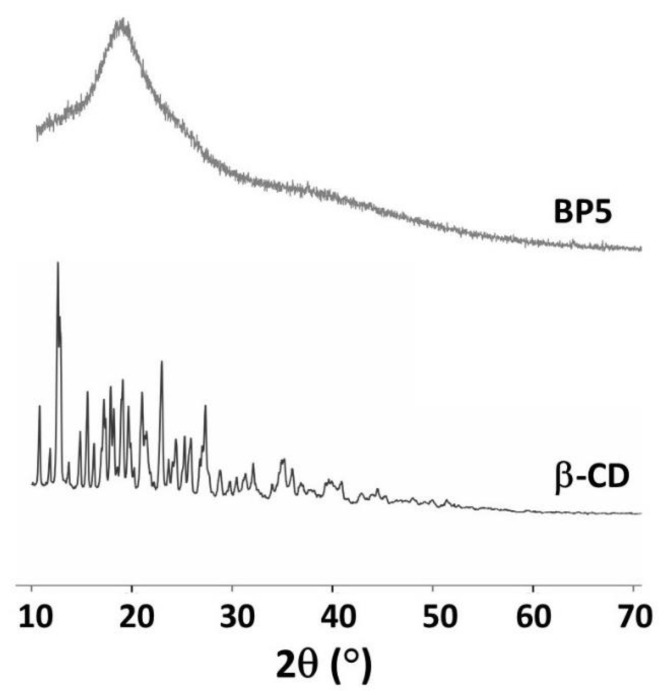
XRD spectra of β-CD and BP5 nanosponges.

The XRD spectra of β-CD and BP5 are presented in Figure 6. The intense and sharp peaks of β-CD indicate a semi-crystalline structure that was indexed to a monoclinic symmetry, as reported in the literature [139]. However, the appearance of a diffraction peak at 18.1° was seen because of the amorphous structure of BP5 nanosponges which confirmed the polymerization between BTCA and β-CD or BTCA and PVOH, leading to destroy the crystallinity of the β-CD and increase the adsorption performance toward pollutants. This result was in accordance with the data reported in a previous study, which informed that the broad peak of CTR cross-linked β-CD nanosponges [109].

### 3.2. Adsorption Study

#### 3.2.1. Preliminary Adsorption Study

The specific properties of both adsorbate and adsorbent, which depended on the pH of the contaminant solutions, played an important role in the adsorption performance such as β-CD (18 g/L of solubility in water at 25 °C), PQ (pH-independent and 620 g/L of solubility in water at 25 °C [21]), SO (a pKa value of 5.8 and 50 g/L of solubility in water at 20 °C [140], and MG (a pKa value of 6.9 [62] and 40 g/L of solubility in water at 25 °C [141]).

The adsorption mechanisms were proposed in four conceivable ways as shown in Figure 1: (i) electrostatic interaction between the anionic charge of BTCA and the cationic charge of cationic contaminants, (ii) host-guest interaction by the formation of inclusion complex which contaminant molecules was encapsulated into the β-CD cavity, (iii) polymeric framework capture which contaminant species were imprisoned in the cross-linked structure (both BTCA cross-linked PVOH and BTCA cross-linked β-CD), and (iv) hydrogen bonding between hydrogen atoms of hydroxyl groups from PVOH and nitrogen atoms from organic pollutant molecules [142].

**Figure 7:**
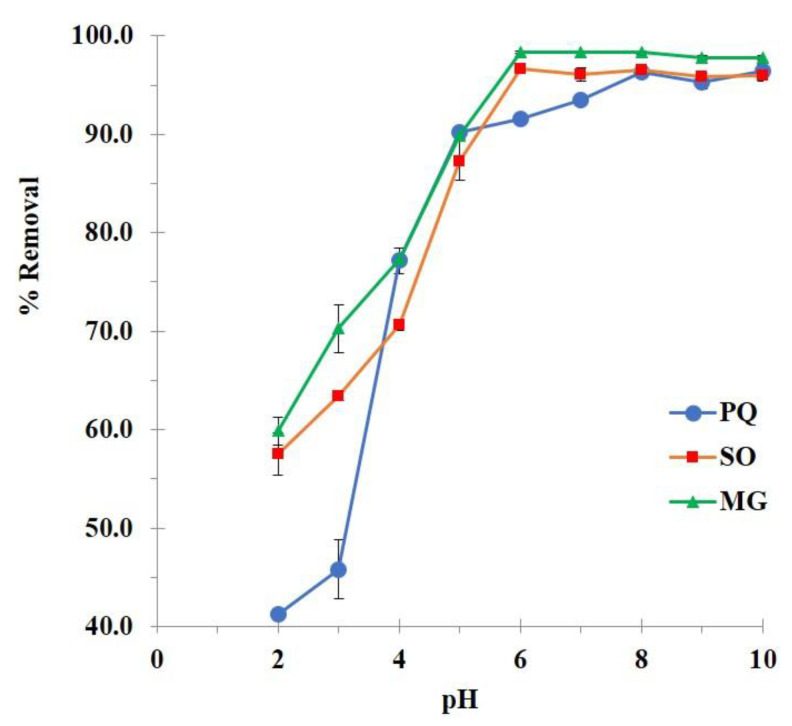
Influence of pH on the contaminant removal (conditions: 2 g/L of adsorbent dosage, 25 mg/L of initial concentration, 180 minutes of contact time, and temperature at 303 K).

The optimization of pH was initially studied as shown in Figure 7. The BP5 nanosponges displayed a low adsorption efficiency at pH 2 (41.2%, 57.5%, and 59.5% of contaminant removal for PQ, SO, and MG adsorption, respectively), which probably happened according to the host–guest interaction, polymeric framework capture, and hydrogen bonding. At this stage, the activation of carboxylate functions from carboxylic groups was restrained because the pH of the solution was smaller than the pKa of BTCA (3.43, 4.58, 5.85, and 7.16). Therefore, the adsorption performance was not good enough due to the lack of electrostatic interaction. The adsorption of nanosponges was then gradually enhanced with pH until reaching the maximum at a pH of 6, 6, and 8 for PQ, SO, and MG adsorption, respectively because of the electrostatic interaction. At this stage, the appearance of carboxylate functions from BTCA could react with cationic charges of contaminant molecules because the pH of the solution was higher than the pKa of BTCA. By the way, the previous study focused on the adsorption of heavy metals, polycyclic aromatic hydrocarbons, and alkylphenols by insoluble polymers issued from hydroxypropyl-β-cyclodextrin cross-linked with BTCA which displayed a point of zero charges (PZC) values of non-activated polymers about 3.4 using titration with the salt addition method [137]. Even though PVOH was added to our nanosponges but the presence of BTCA characters could be explained by this similar work. At pH 2, the surface charge of nanosponges was positive (if pH < PZC) which provided the low adsorption efficiency because hydrogen ions competed dominantly over cationic contaminants and the presence of anionic charge on the surface was very poor. At higher pH (more than 3.4), the surface charge became more and more negative (if pH > PZC), which could progressively adsorb cationic contaminants.

A pKa value of contaminants was also considered for adsorption efficiency. PQ is pH-independent which the adsorption efficiency relied on nanosponges character with an optimal pH of 8. However, SO and MG had a pKa value (5.8 and 6.9, respectively) which these two molecules displayed positive charges by protonation with a pH of the solution below its pKa value. At this circumstance of pH of 6, the nanosponges represented negative charges by carboxylate forms which reacted more favorably with contaminants by electrostatic interaction.

Consequently, these values of pH solution (pH of 6, 6, and 8 for PQ, SO, and MG adsorption, respectively) were also chosen for the next experiment.

**Figure 8:**
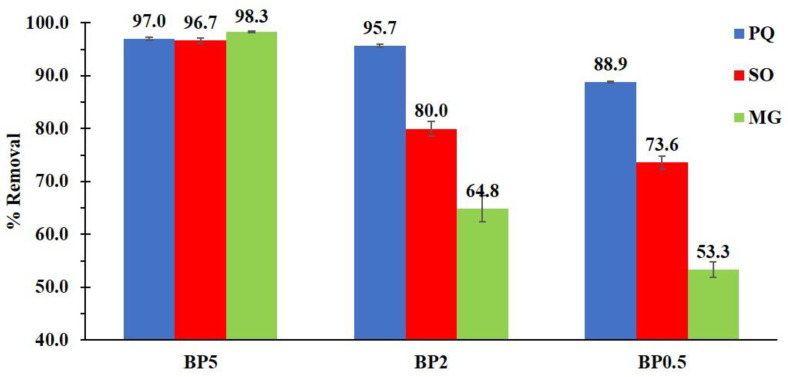
Influence of an amount of PVOH on the contaminant removal (conditions: 2 g/L of adsorbent dosage, optimal pH, 25 mg/L of initial concentration, 180 minutes of contact time and temperature at 303 K).

The BP0.5, BP2, and BP5 nanosponges were subjected to an adsorption experiment to optimize the influence of a quantity of PVOH on adsorption efficiency. Although the increase in PVOH in nanosponges decreased the ion exchange capacity, it demonstrated the contaminant removal significantly as seen in Figure 8. The presence of a higher quantity of PVOH could display dominantly the adsorption performance because of the supplementary interaction via hydrogen bonding from PVOH and contaminants and the network capture of contaminants in the reticulated structure (BTCA cross-linked PVOH). The adsorption efficiency of BP5 nanosponges towards PQ, SO, and MG molecules were 97.0%, 96.7%, and 98.3% respectively which were superior to BP2 and BP0.5, compared to each pollutant. These results were in agreement that informed in the literature, the addition of PVOH in cyclodextrin polymers also enhanced the quantity of aniline extraction [143]. Hence, the presence of PVOH in nanosponges is indispensable for the improvement of adsorption capacity towards cationic pollutants, the BP5 nanosponges were selected for the next study.

In this work, the system without β-CD was not been studied to compare the efficiency of cationic contaminant removal because we reported in the previous work for similar nanosponges using citric acid as a cross-linking agent which had displayed the lower adsorption of PQ for all nanosponges without β-CD, comparing with the system containing β-CD [120].

**Figure 9:**
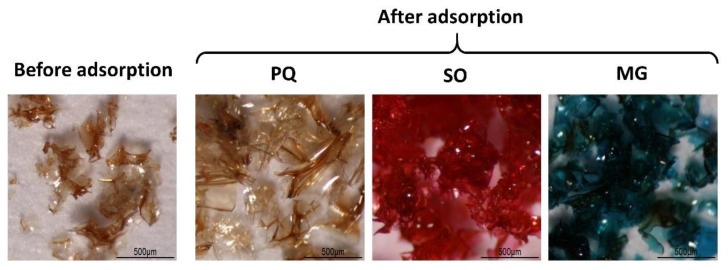
Physical appearance of nanosponges before and after contaminant adsorption.

The physical properties of BP5 nanosponges before and after PQ, SO, and MG, respectively, adsorption was observed is monitored in Figure 9. Before adsorption, BP 5 nanosponges displayed a brown shine transparent flake. Then, samples were swollen with the same color after PQ adsorption, with the red color after SO adsorption, and with the blue-green color after MG adsorption.

#### 3.2.2. Kinetics Study

**Figure 10:**
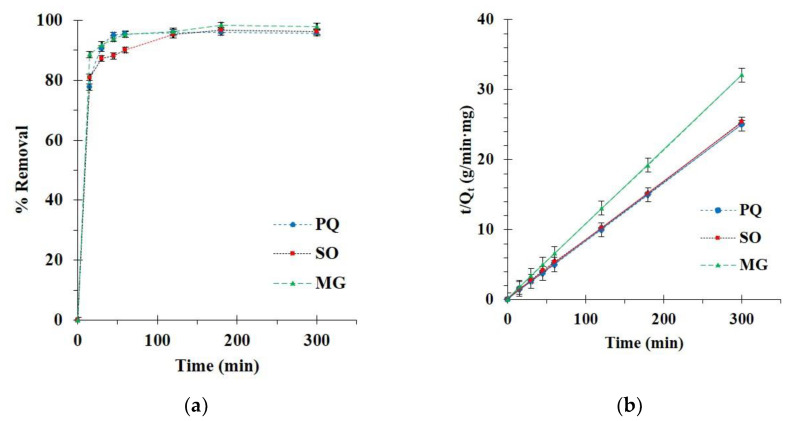
(**a**) Influence of contact time on adsorption capacity; (**b**) Pseudo-second-order kinetics of contaminant adsorption (conditions: 2 g/L of adsorbent dosage, 25 mg/L of PQ initial concentration, optimal, and temperature at 303 K).

The kinetics of BP5 nanosponges towards contaminants was executed with various contact times. The adsorption enhanced remarkably for the initial 15 min until achieving saturation of adsorption after 180 min after adsorption as seen in Figure 10a because of the lack of unoccupied active sites. Thus, a contact time of 180 min was selected for the next study.

The pseudo-first-order and -second-order models were applied to the experimental data in order to understand the adsorption process relating to an adsorption order, chemical reaction, and mass transfer. As observed in Table 2, the correlation coefficients (R^2^) were superior for the pseudo-second-order model (R^2^ = 0.9999, 0.9997, and 0.9999) than the pseudo-first-order model (R^2^ = 0.8097, 0.8727, and 0.9233 for PQ, SO, and MG adsorption, respectively.

The correlation coefficient of the pseudo-second-order model was close to 1 and this model displayed a straight line, which confirmed the suitability of the model with experimental data, as shown in Figure 10b. Therefore, the adsorption capacity was at equilibrium, calculated by the pseudo-second-order model (Q_e,cal_ = 12.0, 11.9, and 9.4 mg/g for PQ, SO, and MG adsorption, respectively).

The intra-particle diffusion model described the diffusion mechanism during adsorption, which was divided into two sections. The fast adsorption with a high adsorption rate constant for the first step (k_31_) was attributed to the boundary layer diffusion. Then, the slow adsorption with a low adsorption rate constant for the first step (k_32_) corresponded to the intraparticle diffusion. The curve of the two parts did not pass through the origin, which confirmed that organic contaminant adsorption was a complicated process [144,145].

#### 3.2.3. Isotherm Study

**Figure 11:**
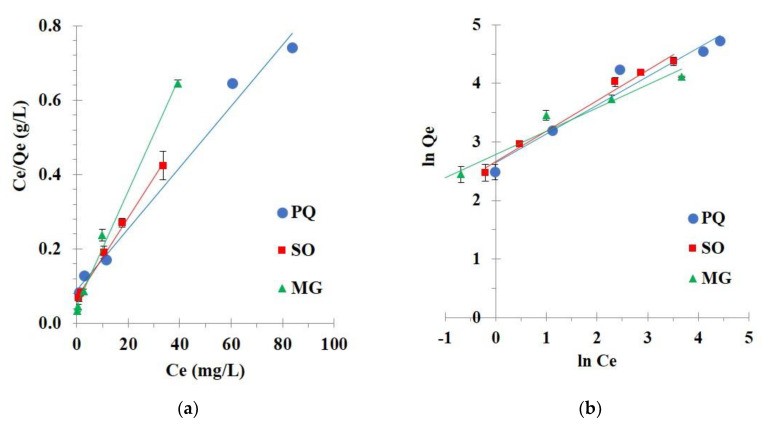
(**a**) Langmuir isotherm; (**b**) Freundlich isotherm of contaminant adsorption (conditions: 2 g/L of adsorbent dosage, 180 minutes of contact time, optimal pH, and temperature at 303 K).

Different initial concentrations of contaminants from 25 to 300 mg/L were investigated for an isotherm study at 30 °C to comprehend the interaction between nanosponges and contaminants at equilibrium state. The Langmuir (Figure 11a) and Freundlich (Figure 11b) isotherm models were applied to the experimental data to evaluate the linearity of these models.

Isotherm parameters were calculated and described in Table 3. The correlation coefficient (R^2^) was essentially superior for the Langmuir isotherm model (R^2^ = 0.9867, 0.9964, and 0.9927) than the Freundlich isotherm model (R^2^ = 0.9486, 0.9854, and 0.9602) for PQ, SO, and MG adsorption, respectively. Langmuir isotherm model displayed a correlation coefficient close to 1 which informed a linear relationship, confirmed the suitability of the model with experimental data, and explained the monolayer adsorption for contaminants on the regular surface of nanosponges. The Chi-square values for the Langmuir model (0.5, 1.9, and 0.2) were lower than the Freundlich isotherm model (6.7, 25.9, and 6.9) for PQ, SO, and MG adsorption, respectively. The small Chi-square values inferred that the experimental data were appropriate with the Langmuir isotherm. Finally, the separation factor (R_L_) was reduced with the rise of initial concentrations for all contaminants, which displayed a strong affinity between nanosponges and contaminants if 0 < R_L_< 1.

In Table 4, the maximum adsorption capacity by cyclodextrin nanosponges was equal to 120.5, 64.9, and 92.6 mg/g for PQ, SO, and MG adsorption, respectively. The adsorption of PQ was good, compared with other adsorbents. However, the removal of SO and MG were mediocre, compared with other adsorbents. Although, the removal efficiency was quite medium. These eco-friendly nanosponges could reuse many times using a washable solvent and it could be applied in a dynamic method or semi-pilot scale to evaluate the adsorption performance.

#### 3.2.4. Reusability Study

**Figure 12:**
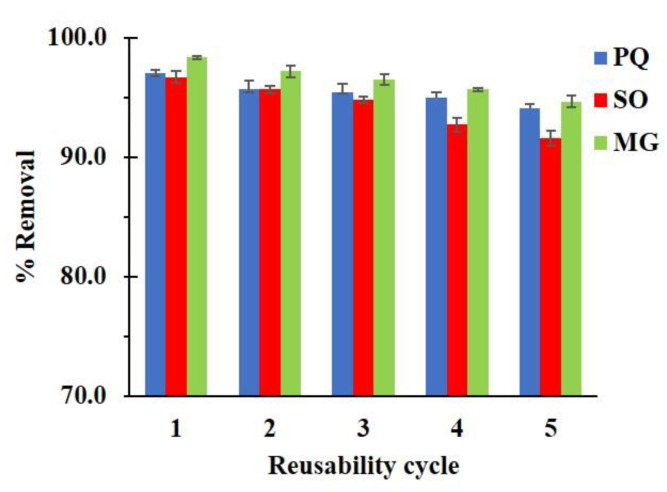
Reusability of C10D-P2 nanosponges (conditions: 2 g/L of adsorbent dosage, pH of 6.5 and temperature at 303 K).

The regeneration of nanosponges was carried out to evaluate the cost-effectiveness of the adsorption process and the usability of nanosponges. In Figure 12, the reusability efficiency reduced slightly after five cycles and attained 94.1%, 91.6%, and 94.6%. Moreover, the contaminant solution could be treated by different methods such as the Fenton reaction, oxidation, photocatalytic degradation, or other convenient methods before releasing and the reuse solution could be separate from other solvents before recycling.

## 4. Conclusions

The preparation of anionic nanosponges was achieved by cross-linking between 1,2,3,4-butanetetracarboxylic acid and β-cyclodextrin in the presence of poly(vinyl alcohol). The BP5 nanosponges resulted in a 68.4% yield, 3.31 mmol/g COOH groups, 0.16 mmol/g β-CD content, 97.0% PQ removal, 96.7% SO removal, and 98.3% MG removal for 25 mg/L of initial concentration. Various characterization techniques confirmed the physicochemical properties of the nanosponges. The adsorption of cationic organic contaminants on cyclodextrin nanosponges happened with four possibilities: (i) an electrostatic interaction between the anionic charge of BTCA and the cationic charge of contaminants, (ii) a host–guest interaction by encapsulation of contaminant molecules into the β-CD cavity, (iii) polymeric framework capture by imprisonment of contaminant molecules in the cross-linked structure, and (iv) hydrogen bonding between hydrogen atoms of PVOH and nitrogen of organic pollutant molecules. Therefore, these nanosponges showed an anionic character with the supplementary function of PVOH to remove cationic contaminants from wastewater. The pseudo-second-order model and the Langmuir isotherm were suitable for kinetics and isotherm study, respectively. The maximum adsorption was 120.5, 92.6, and 64.9 mg/g for PQ, SO, and MG adsorption, respectively. Finally, the reusability performance times achieved 94.1%, 91.6%, and 94.6% for PQ, SO, and MG adsorption, respectively, after five recyclability times. These eco-friendly nanosponges could be a potential green adsorbent to remove cationic organic pollutants that contaminate water.

## Figures and Tables

**Table 1:** Physicochemical properties of cyclodextrin nanosponges and their derivatives.

Name	Composition (% *w*/*v*)			% Yield		IEC (mmol/g)		% Swelling		β-CD Content (mmol/g)	
	β-CD	BTCA	PVOH	Mean	S.D.	Mean	S.D.	Mean	S.D.	Mean	S.D.
BP0.5	10	11.14	0.5	73.3	1.7	3.69	0.05	65.3	1.0	0.085	0.001
BP2	10	11.14	2	71.5	1.8	3.55	0.03	59.1	1.2	0.097	0.006
BP5	10	11.14	5	68.4	1.1	3.31	0.09	54.2	0.5	0.160	0.003

**Table 2:** Pseudo-second-order and pseudo-first-order kinetics parameters (conditions: 2 g/L of adsorbent dosage, 25 mg/L of initial concentration, optimal pH, and temperature at 303 K).

	Q_e (exp)_	Pseudo-First-Order			Pseudo-Second-Order					Adsorption Mechanism	
		R^2^	Q_e (cal)_	k_1_	R^2^	Q_e (cal)_	k_2_	h	t_1/2_	k_31_	k_32_
PQ	12.0	0.8097	1.8	0.0173	0.9999	12.0	0.1427	20.7	0.6	0.5795	0.0045
SO	11.9	0.8727	7.5	0.0041	0.9997	11.9	0.0994	14.2	0.8	0.2757	0.0016
MG	9.4	0.9233	8.4	0.0150	0.9999	9.4	0.1152	10.2	0.9	0.1643	0.0221

**Table 3:** Langmuir and Freundlich isotherm parameters (conditions: 2 g/L of adsorbent dosage, 180 minutes of contact time, pH of 6.5 and temperature at 303 K).

	Q_m (exp)_	Langmuir Isotherm									Freundlich Isotherm				
		R^2^	Q_m (cal)_	K_L_	χ^2^	R_L_ for C_0_ (mg/L)					R^2^	Q_m (cal)_	K_f_	1/n	χ^2^
						25	50	150	250	300					
PQ	112.9	0.9867	120.5	0.09	0.5	0.300	0.176	0.067	0.041	0.034	0.9486	143.9	14.1	0.49	6.7
SO	79.3	0.9964	92.6	0.16	1.9	0.201	0.112	0.040	0.025	0.021	0.9854	139.5	14.4	0.52	25.9
MG	61.1	0.9927	64.9	0.34	0.2	0.107	0.056	0.019	0.012	0.010	0.9602	83.4	16.3	0.40	6.0

**Table 4:** Langmuir isotherm for contaminant removal by various adsorbents.

Contaminant	Adsorbent	Adsorption Dosage	Paraquat Concentration (mg/L)	Maximum Adsorption Capacity
PQ	BTCA cross-linked CD and PVOH nanosponges [This work]	0.02 g in 0.01 L	25–300 mg/L	120.5 mg/g
	Citric acid cross-linked CD and PVOH nanosponges [120]	0.02 g in 0.01 L	25–300 mg/L	112.2 mg/g
	BTCA cross-linked CD nanosponges [21]	0.02 g in 0.01 L	10–200 mg/L	26.7 mg/g
	Citric acid cross-linked CD nanosponges [20]	0.02 g in 0.01 L	10–200 mg/L	21.9 mg/g
	Magnetic adsorbent [24]	0.0025 g in 0.005 L	30–900 mg/L	242.2 mg/g
	Carbon nanotubes [17]	0.002 g in 0.005 L	70–250 mg/L	218.6 mg/g
	Graphene oxide [22]	0.02 g in 0.025 L	4–24 mg/L	29.15 mg/g
	Bentonite [13]	0.04 g in 0.025 L	4–24 mg/L	11.75 mg/g
SO	BTCA cross-linked CD and PVOH nanosponges [This work]	0.02 g in 0.01 L	25–250 mg/L	92.6 mg/g
	Magnetic adsorbent [87]	0.01 g in 0.04 L	50–500 mg/L	769.23 mg/g
	Carbon nanotubes [79]	0.02 g in 0.01 L	1–15 mg/L	8.42 mg/g
	Graphene oxide [84]	0.01 g in 0.02 L	50–1800 mg/L	279.6 mg/g
	Bentonite [76]	0.025 g in 0.05 L	100–600 mg/L	294.1 mg/g
MG	BTCA cross-linked CD and PVOH nanosponges [This work]	0.02 g in 0.01 L	25–250 mg/L	64.9 mg/g
	Magnetic adsorbent [60]	0.02 g in 0.04 L	50–150 mg/L	512.8 mg/g
	Carbon nanotubes [50]	0.02 g in 0.02 L	10–50 mg/L	11.76 mg/g
	Graphene oxide [57]	-	-	102.4 mg/g
	Bentonite [45]	1 g in 0.1 L	50–300 mg/L	178.6 mg/g

## Data Availability

The study did not report any data.
